# Development of matrix metalloproteinase-targeted probes for lung inflammation detection with positron emission tomography

**DOI:** 10.1038/s41598-018-19890-1

**Published:** 2018-01-22

**Authors:** Naoya Kondo, Takashi Temma, Kazuki Aita, Saeka Shimochi, Kazuhiro Koshino, Michio Senda, Hidehiro Iida

**Affiliations:** 1 Department of Investigative Radiology, National Cerebral and Cardiovascular Center Research Institute, 5-7-1 Fujishiro-dai, Suita, Osaka 565-8565 Japan; 2 Department of Biofunctional Analysis, Osaka University of Pharmaceutical Sciences, 4-20-1 Nasahara, Takatsuki, Osaka 569-1094 Japan; 3 Department of Image-based Medicine, Institute of Biomedical Research and Innovation, 2-2, Minatojima Minamimachi, Chuo-ku, Hyogo 650-0047 Japan

## Abstract

As matrix metalloproteinases (MMPs), especially MMP-9 and MMP-12 are involved in the pathological processes associated with chronic obstructive pulmonary disease (COPD), we developed a novel radiofluorinated probe, ^18^F-IPFP, for MMPs-targeted positron emission tomography (PET). ^18^F-IPFP was designed by iodination of MMP inhibitor to enhance the affinity, and labelled with a compact prosthetic agent, 4-nitrophenyl 2-^18^F-fluoropropionate (^18^F-NFP). As a result, IPFP demonstrated the highest affinity toward MMP-12 (IC_50_ = 1.5 nM) among existing PET probes. A COPD model was employed by exposing mice to cigarette smoke and the expression levels of MMP-9 and MMP-12 were significantly increased in the lungs. Radioactivity accumulation in the lungs 90 min after administration of ^18^F-IPFP was 4× higher in COPD mice than normal mice, and 10× higher than in the heart, muscle, and blood. *Ex vivo* PET confirmed the radioactivity distribution in the tissues and autoradiography analysis demonstrated that accumulation differences in the lungs of COPD mice were 2× higher than those of normal mice. These results suggest that ^18^F-IPFP is a promising probe for pulmonary imaging and expected to be applied to various MMP-related diseases for early diagnosis, tracking of therapeutic effects, and new drug development in both preclinical and clinical applications.

## Introduction

Pulmonary diseases including COPD are major causes of morbidity and mortality worldwide^1,2^. COPD is a complex and heterogeneous condition requiring personalized medicine^3^. COPD is an inflammatory disease caused by long-term inhalation exposure to noxious insults, mainly smoking, and characterized by irreversible airway limitation and accelerated decline of lung function. Currently, COPD is diagnosed through clinical manifestations, spirometry, and anatomic imaging, and is limited by insufficient assessment of physiological alterations. Thus, development of *in vivo* molecular imaging methods is required to contribute to early diagnosis of COPD and evaluation of therapeutic interventions.

MMPs are a family of zinc-dependent proteinases that play important roles in degrading the extracellular matrix and tissue remodeling both in normal physiological states and in abnormal pathological processes. MMPs play a key role in pulmonary inflammation and are recognized as promising diagnostic and therapeutic targets in COPD^4–6^. Among the various subtypes of MMPs, MMP-12, a potent elastase, is an essential mediator of the pathophysiology of COPD^5,7,8^, and MMP-9 functions as an indicator of the clinical COPD stage^9–11^. Recently, a peptidomimetic inhibitor, namely ML5, originally synthesized by Leeuwenburgh *et al*.^12,13^, which consists of a succinylhydroxamate attached to the *N*-terminus of phenylalanine-lysine dipeptide, was reported to show high and moderate binding affinities to MMP-12 and MMP-9, respectively, in a fluorogenic inhibition assay (IC_50_ = 2.0 nM and 19.5 nM for MMP-12 and MMP-9, respectively^14^). Moreover, ML5 is a useful lead compound due to availability of the ε-amino group of the lysine residue for radiolabeling to develop *in vivo* molecular imaging probes. However, ML5 radiolabeled with *N*-succinimidyl 4-^18^F-fluorobenzoate (^18^F-SFB), ^18^F-FB-ML5, exhibited an ~100-fold loss of affinity for MMP-12 compared to the parent compound (IC_50_ = 138.0 nM and 31.5 nM for MMP-12 and MMP-9, respectively^14^), which led to fairly diminished performance in *in vivo* experiments using tumor bearing mice^14^ and short-term CS exposed mice^15^.

**Figure 1:**
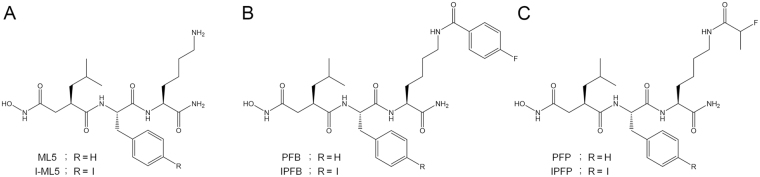
Structures and designs of compounds in this study. (**A**) ML5 (R = H), I-ML5 (R = I) (**B**) PFB (R = H), IPFB (R = I) and (**C**) PFP (R = H), IPFP (R = I).

Herein, we aimed to develop a potent MMP probe, specifically targeting MMP-12 and MMP-9, based on ML5 for noninvasive COPD imaging with PET^16^. As attachment of the ^18^F-fluorobenzoyl moiety decreased the affinity of ML5 toward MMP-12, we selected another prosthetic agent for radiofluorination, namely 4-nitrophenyl 2-^18^F-fluoropropionate (^18^F-NFP)^17^, as its smaller molecular weight may result in weaker steric hindrance toward MMP-12 compared with ^18^F-SFB. In addition, iodination of the benzyl group of the phenylalanine residue in a substrate peptide reportedly functions to improve the affinity toward MMP-9^18^. We synthesized 4 compounds based on ML5 with differences in prosthetic agents and iodine addition (Fig. 1), and compared their respective *in vitro* inhibitory potencies against MMP protease activities in a fluorogenic assay. Finally, we radiofluorinated the most potent compound and evaluated its effectiveness using long-term CS exposed mice, a common COPD model animal^19^, in an *in vivo* biodistribution study, dynamic PET, and *ex vivo* autoradiographic analysis.

## Results

### Synthesis

ML5 and I-ML5 were obtained at yields of 20 and 25%, respectively, while PFB, IPFB, PFP and IPFP were synthesized at yields of 15, 35, 12 and 30%, respectively (Supplemental Table 1) (Fig. 1). The chemical purities of all compounds were estimated to be above 99% from HPLC-UV chromatograms.

### ***In vitro*** affinity and selectivity estimation

The IC_50_ values of ML5, I-ML5, PFB, IPFB, PFP and IPFP toward the various MMPs are summarized in Table 1. ML5 exhibited high affinity for MMP-12 and moderate affinities for MMP-2, MMP-9 and MMP-13, consistent with a previous report^14^. The iodinated compounds (I-ML5, IPFB, and IPFP) exhibited higher affinities toward all MMPs tested than the corresponding deiodinated compounds (ML5, PFB, and PFP). While compounds containing the fluorobenzoyl moiety (PFB and IPFB) revealed significant loss of affinity toward all MMPs, compounds containing the fluoropropyl moiety (PFP and IPFP) sustained the affinity compared with the parent compounds (ML5 and I-ML5). IPFP demonstrated the highest affinity toward all MMPs among the compounds tested, with IC_50_ values of 4.4 ± 2.3, 8.1 ± 2.0, 1.5 ± 0.8, and 4.1 ± 1.7 nM for MMP-2, MMP-9, MMP-12, and MMP-13, respectively. Therefore, IPFP exhibits moderate selectivity toward MMP-12, with inverse IC_50_ ratios of 2.9, 5.4, and 2.7 relative to MMP-2, MMP-9, and MMP-13, respectively.

**Table 1:** IC_50_ (nM) values toward MMPs.

Compound	MMP-2	MMP-9	MMP-12	MMP-13
ML5	9.6 ± 4.7	37.9 ± 5.2	2.8 ± 0.4	22.4 ± 8.1
I-ML5	3.7 ± 1.0	13.8 ± 1.5	2.3 ± 1.1	11.8 ± 6.3
PFB	77.7 ± 4.0	178.0 ± 24.2	24.2 ± 14.1	66.1 ± 26.1
IPFB	29.1 ± 9.3	148.0 ± 30.6	12.3 ± 4.0	44.5 ± 21.6
PFP	12.5 ± 7.1	54.1 ± 38.3	4.5 ± 2.5	11.1 ± 6.6
IPFP	4.4 ± 2.3	8.1 ± 2.0	1.5 ± 0.8	4.1 ± 1.7

### Radiosynthesis

^18^F-NFP was successfully synthesized via a one-step reaction using an automated synthesizer at a radiochemical yield of around 2.5% (decay corrected). ^18^F-IPFP was obtained in no carrier added form via conjugation of ^18^F-NFP to I-ML5 at a radiochemical yield of 58 ± 9.4% (decay corrected) and a radiochemical purity of greater than 99%. Overall radiochemical yield (^18^F-Fluoride to ^18^F-IPFP) was 1.3 ± 0.2% and synthesis time was within 150 min, including reaction time, HPLC purification, solvent evaporation, and formulation for animal experiments. The precursor I-ML5 (retention time = 9.4 min) and ^18^F-IPFP (retention time = 12.0 min) were completely separated by RP-HPLC and the molar activity of ^18^F-IPFP was 1.5 ± 0.2 TBq/mol.

### ***In vivo*** study

**Figure 2:**
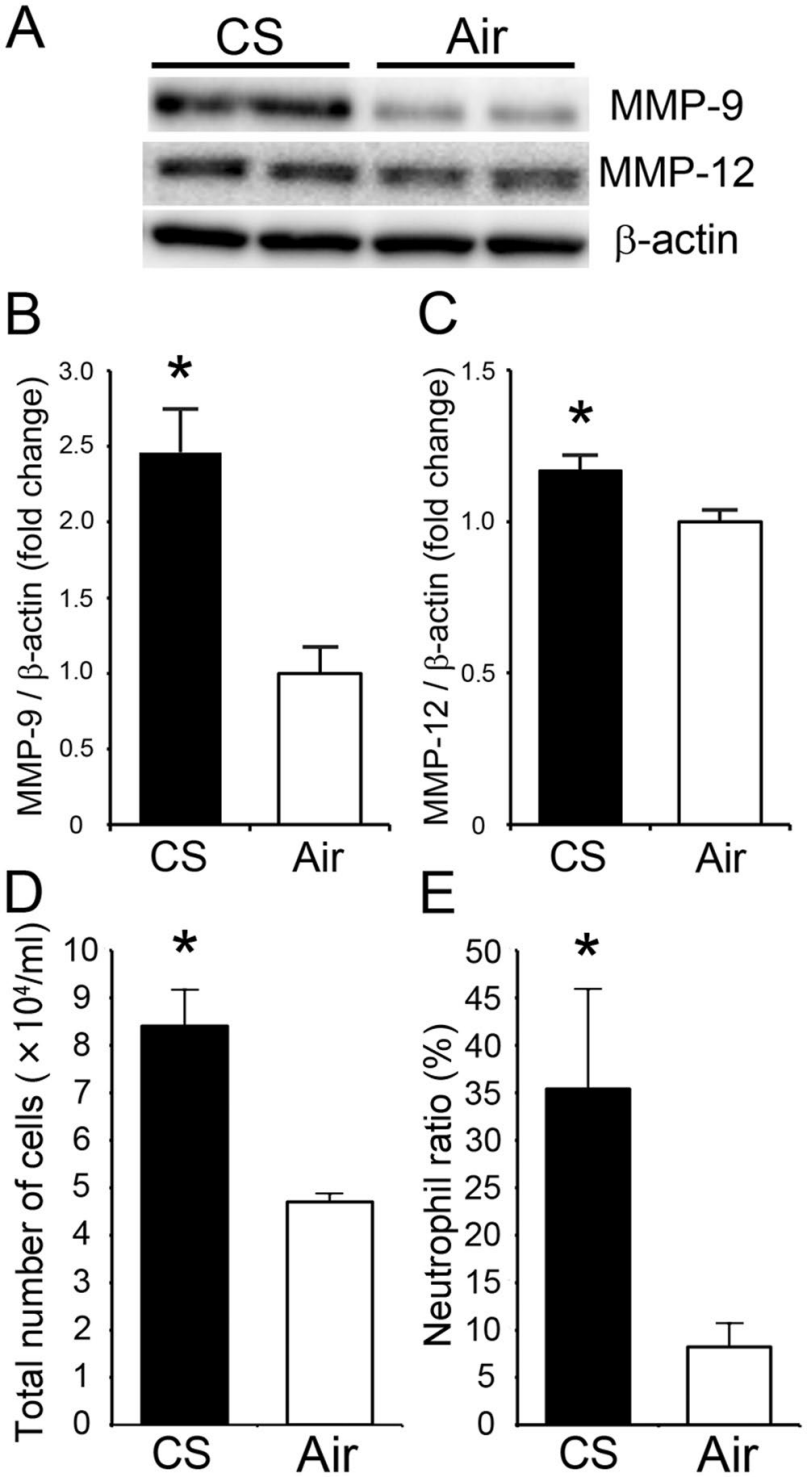
(**A**) Representative western blot bands: MMP-9, MMP-12 and β-actin in lung homogenates of the CS and Air groups from different gels. Full-length gels and blots are shown in a supplementary information (Supplemental Fig. 2) (**B**,**C**) Expression of MMP-9 (**B**) and MMP-12 (**C**) in the CS group compared with the Air group after normalization to β-actin. (**D**) Total cells per 1 ml bronchoalveolar lavage fluid. (**E**) The ratio of neutrophil in bronchoalveolar lavage fluid. *p < 0.05 vs corresponding Air groups.

Expression of MMP-9 (fold change = 2.5) and MMP-12 (fold change = 1.2) was significantly increased in the CS group (Fig. 2A–C). The total number of cells in the bronchoalveolar lavage fluid was higher in the CS group (8.4 × 10^4^/ml) than the Air group (4.7 × 10^4^/ml). The neutrophil ratio in the CS group (35.4%) was also higher than that of the Air group (8.2%) (Fig. 2D,E).

**Figure 3:**
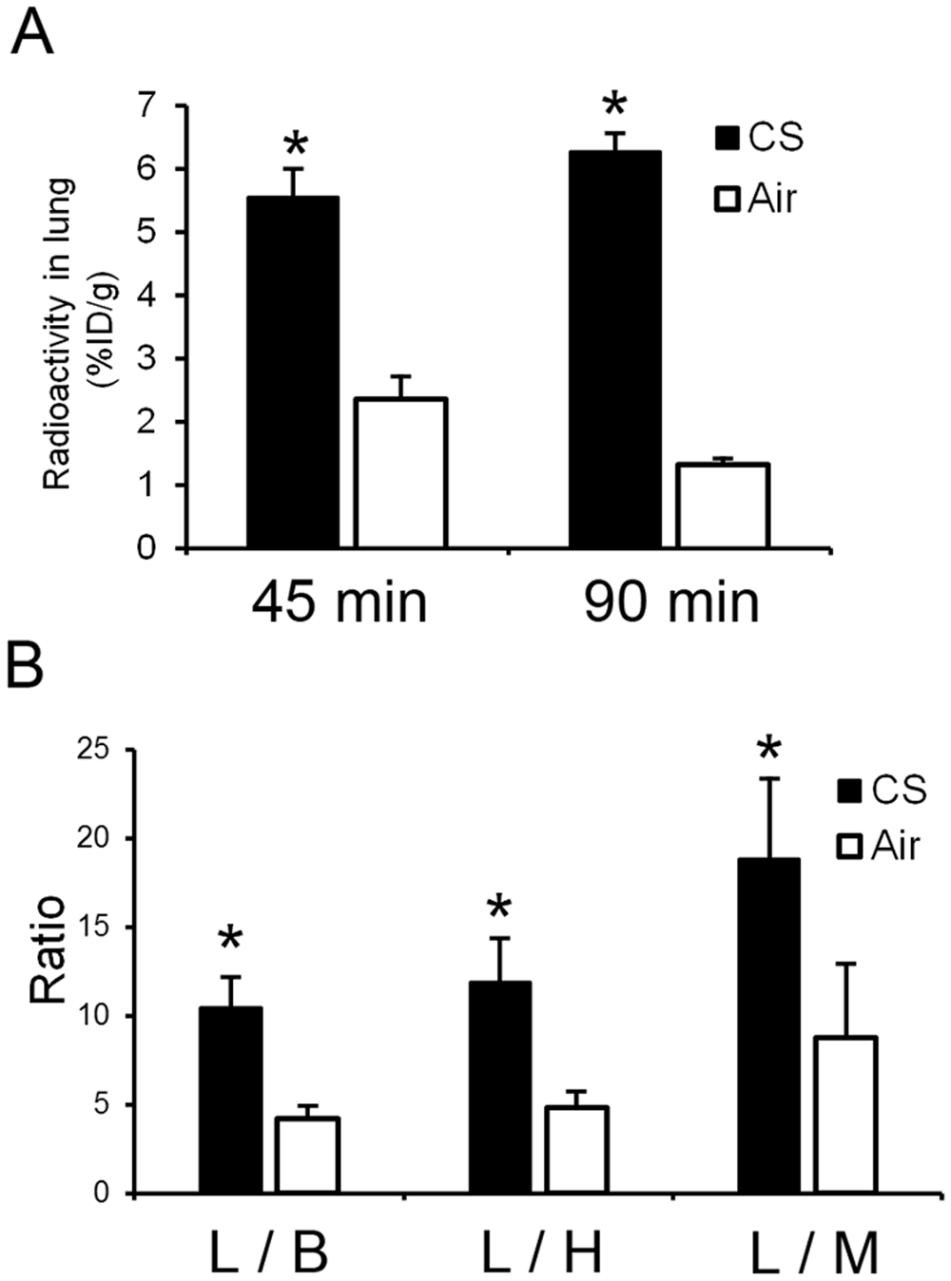
(**A**) Radioactivity accumulation in the lungs of mice in the CS and Air groups at 45 and 90 min after injection of ^18^F-IPFP. (**B**) Radioactivity accumulation ratio (L/B, lung to blood; L/H, lung to heart, L/M, lung to muscle) for the CS and Air groups at 45 min after injection of ^18^F-IPFP. *p < 0.05 vs corresponding Air groups calculated by unpaired t-test with Welch’s correction.

Biodistribution data after ^18^F-IPFP injection are reported in Tables 2 and 3 for the Air and CS groups, respectively. ^18^F-IPFP rapidly distributed to tissues with low residuals in blood circulation, as indicated by the 0.8 ± 0.1% injected dose per gram (%ID/g) in the blood at 5 min post injection in the CS group. The CS and Air groups showed similar radioactivity distribution profiles in most tissues evaluated except for the lungs; the CS group revealed more than a 4-fold higher radioactivity accumulation in the lungs (6.3 ± 0.3%ID/g at 90 min) compared with the Air group (1.3 ± 0.1%ID/g at 90 min) (Fig. 3A). Furthermore, the CS group exhibited more than a 10-fold higher radioactivity accumulation in the lungs (5.6 ± 0.5%ID/g) at 45 min after injection than surrounding tissues: 0.5 ± 0.1%ID/g in the heart, 0.5 ± 0.1%ID/g in the blood, and 0.3 ± 0.1%ID/g in the muscle (Fig. 3B). Radioactivity uptake in the bone was low over the time window, suggesting the absence of defluorination after injection.

**Table 2:** Biodistribution of radioactivity after ^18^F-IPFP injection in the Air group^§^.

	Time after injection (min)		
	20	45	90
Blood	0.5 ± 0.1	0.6 ± 0.0	0.7 ± 0.1
Heart	0.5 ± 0.1	0.5 ± 0.0	0.5 ± 0.1
Lung	5.3 ± 0.6	2.4 ± 0.4	1.3 ± 0.1
Liver	22.6 ± 0.7	15.2 ± 1.6	11.2 ± 1.1
Kidney	37.6 ± 6.4	38.8 ± 4.2	34.6 ± 3.3
Stomach^¶^	2.8 ± 2.1	1.5 ± 1.0	0.5 ± 0.2
Intestine	10.7 ± 2.2	16.9 ± 3.0	18.4 ± 2.1
Pancreas	5.4 ± 0.7	4.5 ± 0.8	3.9 ± 0.6
Spleen	0.9 ± 0.1	0.8 ± 0.1	0.8 ± 0.2
Muscle	0.3 ± 0.1	0.3 ± 0.2	0.4 ± 0.1
Bone	0.4 ± 0.0	0.5 ± 0.1	0.7 ± 0.2

^§^Tissue radioactivity is expressed as % injected dose per gram. ^¶^Expressed as % injected dose.

**Table 3:** Biodistribution of radioactivity after ^18^F-IPFP injection in the CS group^§^.

	Time after injection (min)		
	5	45	90
Blood	0.8 ± 0.1	0.5 ± 0.1	0.6 ± 0.1
Heart	0.9 ± 0.3	0.5 ± 0.1	0.6 ± 0.1
Lung	9.4 ± 1.9	5.6 ± 0.5	6.3 ± 0.3
Liver	30.8 ± 4.6	17.4 ± 4.1	12.0 ± 1.0
Kidney	34.1 ± 3.6	31.7 ± 2.2	25.4 ± 5.2
Stomach^¶^	1.0 ± 0.4	2.2 ± 0.2	1.4 ± 0.5
Intestine	6.0 ± 0.6	13.1 ± 0.3	15.7 ± 0.9
Pancreas	6.2 ± 0.3	4.8 ± 0.6	3.9 ± 0.4
Spleen	2.5 ± 0.4	1.2 ± 0.2	1.3 ± 0.2
Muscle	0.6 ± 0.1	0.3 ± 0.1	0.3 ± 0.1
Bone	1.0 ± 0.3	0.6 ± 0.1	0.8 ± 0.2

^§^Tissue radioactivity is expressed as % injected dose per gram. ^¶^Expressed as % injected dose.

### PET experiments

**Figure 4:**
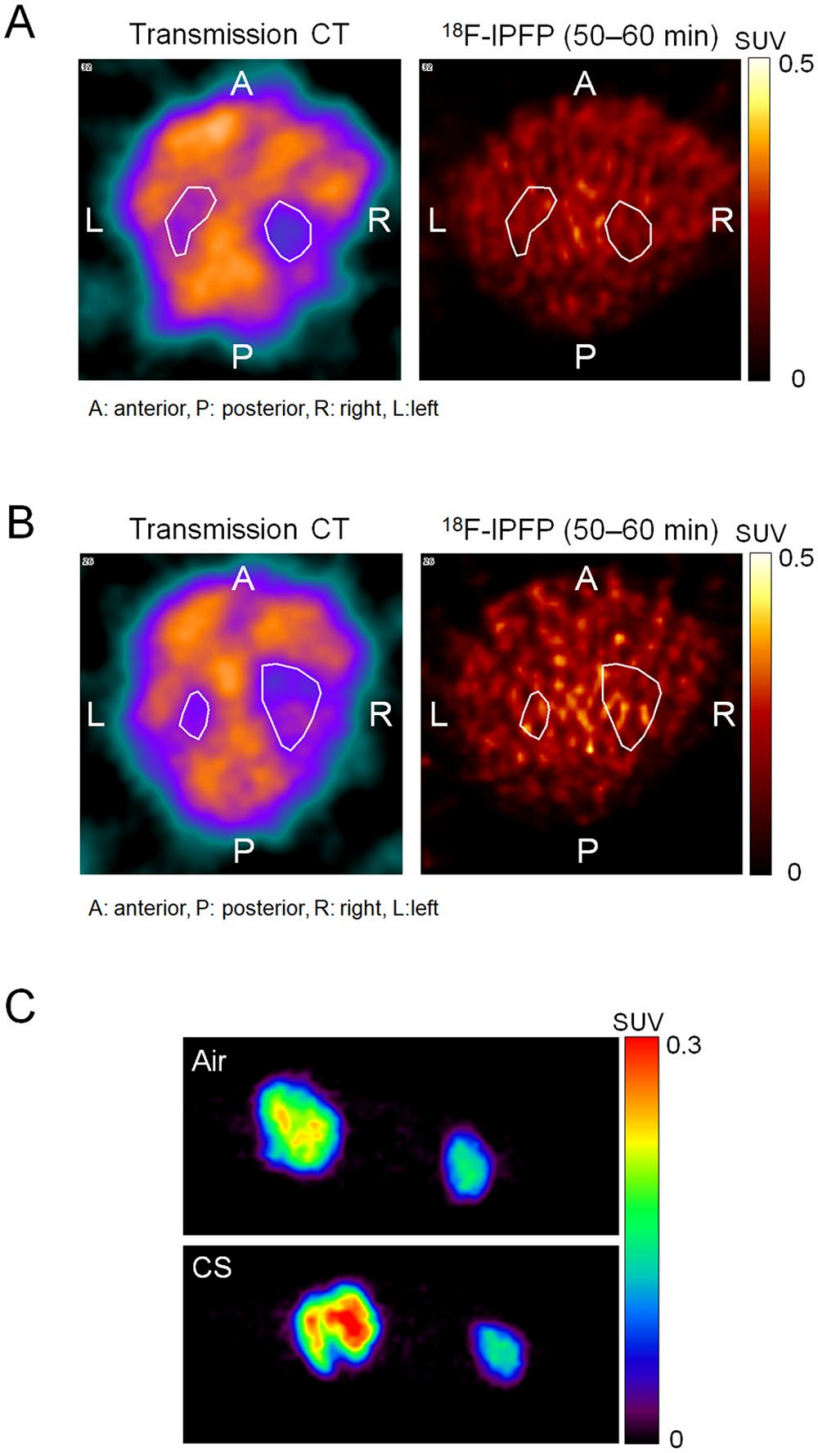
Transverse view of transmission CT and PET images at 50–60 min after injection of ^18^F-IPFP in the (**A**) Air and (**B**) CS groups. (**C**) Maximum intensity projection images of removed lungs (left) and heart (right) from the Air (upper) and CS (lower) groups.

**Figure 5:**
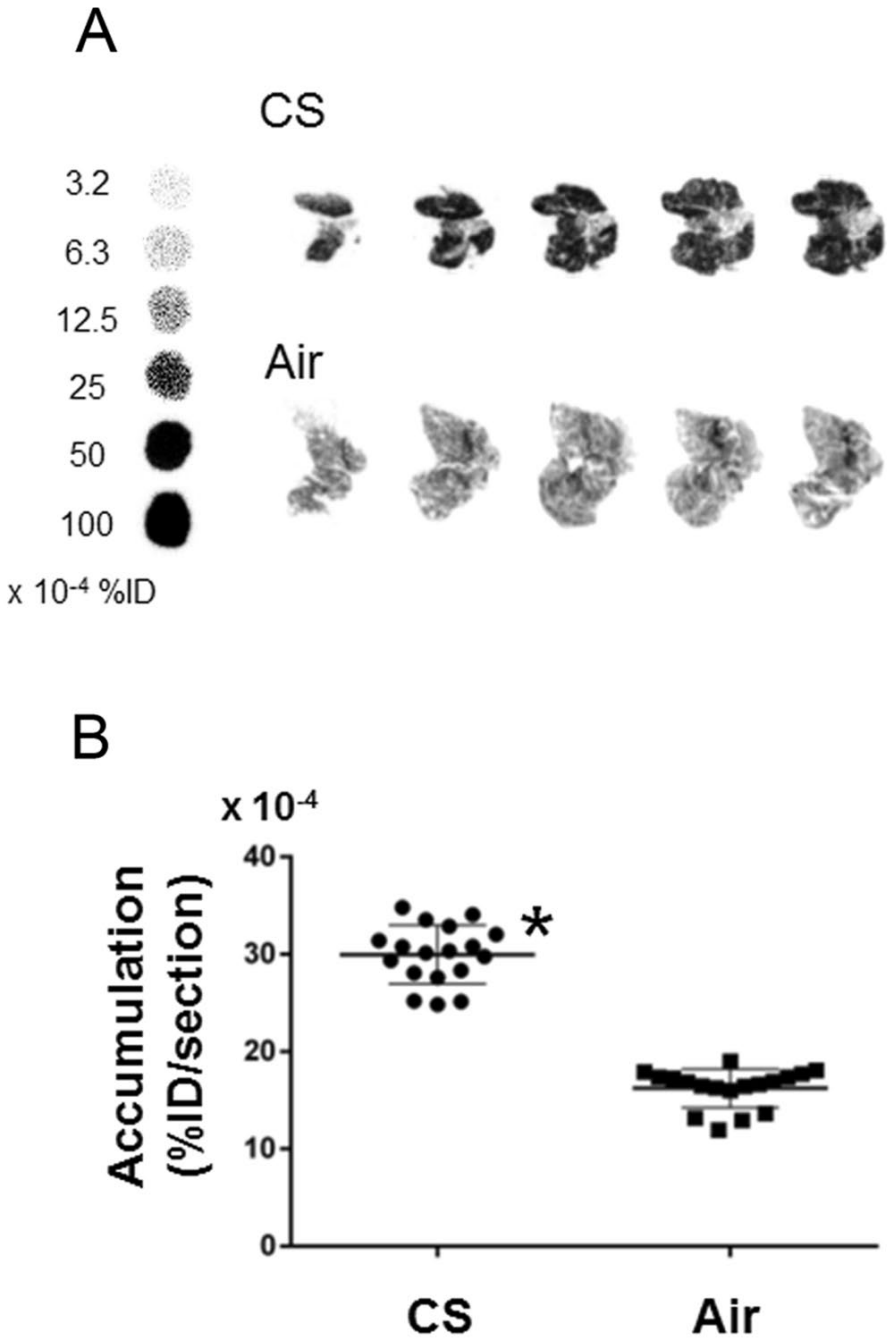
(**A**) Representative autoradiogram sections obtained from the Air and CS groups after the PET scan, together with reference ^18^F-IPFP solutions. (**B**) Calculated radioactivity concentration (%ID/section) in CS and Air groups (n = 18, each). *p < 0.0001 vs Air group.

*In vivo* PET images obtained 50–60 min after injection of ^18^F-IPFP showed that radioactivity accumulation in the lung regions contoured by the transmission CT images were hardly detectable in both the Air (SUV; 0.17) and CS (SUV; 0.22) groups (Fig. 4A,B). Maximum intensity projection images of removed lungs and hearts showed that the lungs could be detected and exhibited greater radioactivity accumulation than the heart in both study groups, as well as greater accumulation in the CS group (Fig. 4C). Autoradiograms obtained after PET imaging showed a homogeneous accumulation profile in lung tissues (Fig. 5A). Quantitation of the accumulated radioactivity data in the lung sections at intervals of 100 μm suggests that ^18^F-IPFP is entirely distributed in lungs, with about a 2-fold higher radioactivity accumulation in the CS group than the Air group (30.0 ± 3.0 × 10^−4^ vs. 16.2 ± 2.0 × 10^−4^%ID/section, *p* < 0.0001) (Fig. 5B).

**Figure 6:**
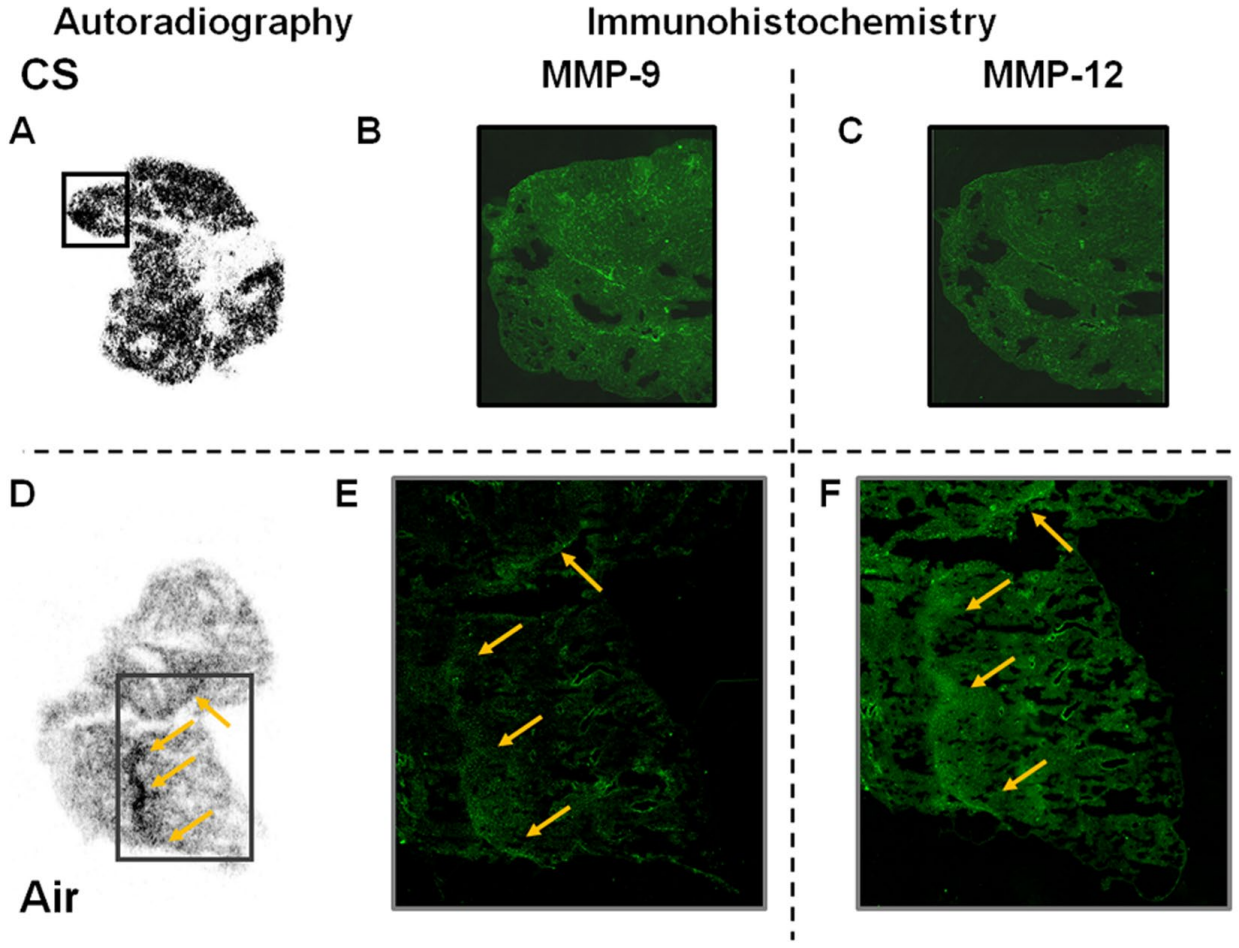
Representative autoradiogram sections obtained from the (**A**) CS and (**D**) Air groups after the PET scan and immunohistochemistry of (**B**) MMP-9 and (**C**) MMP-12 expression in CS group and representative sections of (**E**) MMP-9 and (**F**) MMP-12 expression in Air group. The yellow arrows indicate colocalization of MMPs expression and ^18^F-IPFP accumulation.

Fluorescent immunostaining showed stronger MMP-9 expression in CS group than Air group (Fig. 6B and E). On the other hand, there was a little difference in the MMP-12 expression between CS and Air groups (Fig. 6C and F). In CS group, expression of MMPs were increased and almost uniformly observed (Fig. 6B,C). This is consistent with the results of autoradiograms where ^18^F-IPFP is highly accumulated over all in the lungs (Fig. 6A). On the other hand, in the Air group, localization of MMPs expression was observed (Fig. 6E and F), and this localization was also consistent with the localization of ^18^F-IPFP found in an autoradiogram (Fig. 6D).

## Discussion

Herein, we developed a novel PET imaging probe, namely ^18^F-IPFP, based on the structure of an MMP inhibitor and examined the feasibility of *in vivo* MMP-targeted pulmonary imaging in a COPD mouse model. To the best of our knowledge, ^18^F-IPFP exhibited the highest affinity for MMP-12 among probes that have been reported to date, and demonstrated sufficiently high affinities for MMP-9, MMP-2 and MMP-13. As MMP-12 is an essential mediator of the pathophysiology of COPD in human patients, and MMP-9 is an indicator of clinical COPD stage, ^18^F-IPFP is expected to provide an effective means for diagnosis of COPD. The effectiveness of the probe was also confirmed from the significantly high radioactivity accumulation in the lungs of COPD mice treated with ^18^F-IPFP.

As the current diagnosis of COPD is based on spirometry and anatomical imaging, functional imaging has been required for assessment of disease progression and evaluation of treatment. ^18^F-FDG is typically used for monitoring inflammation, specifically neutrophilic recruitment, in COPD patients. However, ^18^F-FDG also accumulates in macrophages, lymphocytes, and eosinophils, as well as structural cells within the lungs, so that increased ^18^F-FDG uptake in the lungs reflects the integrated inflammatory response and cannot be used as a specific indicator of inflammation^20^. In contrast, MMPs are known to be diagnostic markers of COPD, and MMP inhibitors have been shown to be effective for COPD treatment in preclinical experiments^4–6^. Therefore, *in vivo* imaging of MMP expression could contribute to the diagnosis and treatment of COPD.

We developed an ^18^F-labeled PET imaging probe for imaging MMP-12 and MMP-9 based on the inhibitor ML5 using ^18^F-NFP as a prosthetic agent, in contrast to a previous report using ^18^F-SFB^14^. Compared with ^18^F-SFB, ^18^F-NFP provides a smaller radioactive moiety, without an aromatic ring, to the inhibitor, so that the labeling procedure has fewer effects on the resulting properties of the inhibitor, such as affinity, specificity, and pharmacokinetics. In fact, our fluorogenic inhibition assay revealed that ^18^F-SFB labeling markedly reduced the affinities of labeled compounds to MMPs^14^, whereas ^18^F-NFP labeling maintained the affinities (Table 1). In addition, incorporation of iodine into the labeled compound also contributed to improving the affinity of the probes. We initially expected that iodine incorporation would only improve the affinity for MMP-9, but, instead, it resulted in the improvement of affinities for all the MMPs tested. This may be due to the interaction of iodine not only with the binding pocket S1’^18^, but also with a common structure of the MMPs. Further studies, such as a molecular modeling analysis of the interaction between IPFP and MMPs, are required to elucidate the detailed contribution of iodine, and may lead to development of MMP-selective probes. Nevertheless, the novel probe, ^18^F-IPFP, obtained from conjugation of I-ML5 with ^18^F-NFP, was found to exhibit the highest affinity for MMP-12 (1.5 nM) and high affinities for MMP-9, MMP-2 and MMP-13 (Table 1).

The COPD mouse model was produced by exposing mice to CS for 4 months. Changes in the expression levels of MMPs in the lungs were confirmed by western blot analysis, and showed expression rates of MMP-9 and MMP-12 similar to those of a previous report that employed 6-month exposure^21^. We also investigated the increase in the number of cells and the percentage of neutrophils in bronchoalveolar lavage fluid, confirming previous results^22^. Biodistribution experiments showed 4 times higher radioactivity accumulation in the lungs of mice in the CS group compared with those in the Air group at 90 min after ^18^F-IPFP administration (Fig. 3). Although MMP-12 is known to be highly expressed in human COPD patients, model mice in the CS group exhibited small changes in MMP-12 expression relative to the Air group (fold change = 1.2), as previously reported^21^, indicating that other MMPs, such as MMP-9 and MMP-2, which are elevated during lung inflammation^21^, contributed to probe accumulation in the lungs of the disease mice. Regarding the accumulation of ^18^F-IPFP, having high affinities for plural MMPs is thought to favor the detection of inflammation.

Radioactivity accumulation in the lungs was higher than in the heart, muscle, and blood, supporting the validity of ^18^F-IPFP for pulmonary imaging with proper pharmacokinetics. However, whole body PET imaging did not delineate the lungs of mice (Fig. 4A,B) due to low tissue density of lungs containing air in the alveoli. The low tissue density made radioactivity accumulation in lung tissue difficult to detect in PET images expressed as radioactivity per unit volume (Bq/ml). Therefore, it may be helpful to compute ratio images, that is, radioactivity images relative to the tissue density images derived from transmission CT images, to detect radioactivity accumulation in lung tissue *in vivo*. In fact, *ex vivo* PET images achieved a clear visualization of the lung and differences in accumulation between normal and COPD lung could be detected (Fig. 4C). Similarly, autoradiography analysis was able to quantify the 2-fold accumulation differences in the CS and Air groups (Fig. 5) and revealed that the radioactivity distribution of ^18^F-IPFP tended to coincide with MMP-9 and MMP-12 expression areas (Fig. 6). These results indicated that ^18^F-IPFP recognized MMPs in lungs *in vivo*. It is also possible that high accumulation in the liver affect the quantification of radioactivity accumulation in case of pulmonary imaging. Although the accumulation ratio of lung to liver was greatly improved (>0.5) in comparison with the previously reported ^18^F-SFB labeled ^18^F-FB-ML5 (<0.1)^15^, ^18^F-IPFP may still need to reduce liver radioactivity accumulation for diagnosis purpose. To reduce the accumulation in liver, it may be effective to lower the lipophilicity of the probes. Further research on image processing, taking into consideration information on tissue density, and using larger animals with optimal respiratory gating^23^, will greatly improve PET images obtained using ^18^F-IPFP.

We demonstrated that ^18^F-IPFP showed potent binding affinity for MMPs, especially MMP-12, and exhibited a promising ability to detect elevation of MMPs *in vivo*. Thus, ^18^F-IPFP is expected to be applied to various MMP-related diseases for early diagnosis and tracking of therapeutic effects. However, further interpretation, such as which MMPs are actually involved in the accumulation, may be necessary for detailed diagnostic purposes. To that end, higher selectivity probes may be required. To develop such highly selective probes in the future, we think further optimization of IPFP, or utilization of MMP inhibitors that exhibit higher selectivities than ML5, may be needed. We believe that the labeling method utilizing ^18^F-NFP demonstrated in this study can be effectively used for such future probe development.

In conclusion, we developed a ^18^F-labeled MMP-targeted probe, ^18^F-IPFP, which was found to exhibit, to the best of our knowledge, the highest affinity toward MMP-12 among probes developed to date, and demonstrated its effectiveness for pulmonary imaging in a COPD mouse model. This study also demonstrated a rational drug design for the development of short peptide-based probes. ^18^F-IPFP is expected to be applied to various MMP-related diseases such as pulmonary inflammation, atherosclerosis, and cancer, for early diagnosis, tracking of therapeutic effects, and new drug development in both preclinical and clinical applications.

## Materials and Methods

### General

All reagents were obtained commercially and used without further purification unless otherwise noted.

### Synthesis

ML5 and an iodinated derivative of ML5 (I-ML5) were synthesized according to a previous report^12^ (Fig. 1A; Supplemental Scheme 1). ML5 and I-ML5 were reacted with SFB according to a previous report, to yield PFB and IPFB, respectively^14^ (Fig. 1B; Supplemental Scheme 2). NFP was synthesized according to a literature procedure^17^ and subsequently reacted with ML5 and I-ML5, following the same procedure as used for PFB, to yield PFP and IPFP, respectively (Fig. 1C; Supplemental Scheme 3). The resulting compounds were purified by reversed-phase high-performance liquid chromatography (RP-HPLC) and characterized by electrospray ionization mass spectrometry and ^1^H NMR recorded by VNMRS NMR System (Agilent, CA, USA) with tetramethylsilane (TMS) as an internal standard.

### ***In Vitro*** Affinity and Selectivity Estimation

Determination of the IC_50_ toward human MMP-2, MMP-9, MMP-12 and MMP-13 was performed by a competitive enzyme activity assay according to a literature procedure^6^. The assay was performed by incubating 5 μM of the synthesized fluorogenic peptide substrate Mca-Pro-Leu-Gly-Leu-Dpa(Dnp)-Ala-Arg-NH2 with 5 nM recombinant human MMP-2 (AS72005, Anaspec Inc., CA, USA), MMP-9 (911-MP, R&D Systems, MN, USA), MMP-12 (917-MP, R&D Systems) and MMP-13 (AS72011, Anaspec) along with various concentrations of ML5, I-ML5, PFB, PFP, IPFB, and IPFP (0.2–365 nM) in a buffer (pH 7.5, 50 mM tris(hydroxymethyl)aminomethane, 10 mM calcium chloride, 150 mM sodium chloride, and 0.01% Brij-35) containing 5% dimethyl sulfoxide. The increase in fluorescence over time was measured using a SpectraMax M2e microplate reader (Molecular Devices Inc., CA, USA) with an excitation wavelength of 325 nm and an emission wavelength of 395 nm. The fluorescence intensity after 30 min was plotted as a function of the concentration of each of the compounds on a logarithmic scale to produce an inhibition curve that was subsequently fit with a sigmoidal function by a least squares method. The IC_50_ values were calculated from the inhibition curves using GraphPad Prism 6 (GraphPad Software, Inc., CA, USA).

### Radiosynthesis

Fluorine-18 was produced as an ^18^F-fluoride ion via the ^18^O(p,n)^18^F reaction in ^18^O-water using an in-house CYPRIS-HM18 cyclotron (Sumitomo Heavy Industries, Ltd., Tokyo, Japan). ^18^F-NFP was synthesized following a previous report with slight modifications^17^ (Supplemental Scheme 4). ^18^F-NFP was concentrated (100–300 MBq/200 μL acetonitrile) and transferred to a solution of I-ML5 (500 μg) in 200 μL phosphate buffer and reacted at 50 °C for 30 min. The reactant was purified with a RP-HPLC system equipped with an RI detector and the collected fraction containing ^18^F-IPFP was concentrated by a vacuum evaporator and then redissolved in saline.

^18^F-IPFP was identified by radio-HPLC co-elution with the non-radioactive IPFP (Supplemental Fig. 1).

### Animal Preparation

Male BALB/c mice (5 weeks old) were purchased from Japan SLC (Shizuoka, Japan) and housed under a 12 h light/12 h dark cycle with free access to food and water. The animal experiments in this study were conducted in accordance with institutional guidelines and approved by the institutional animal care committee of the National Cerebral and Cardiovascular Center.

The animals were randomly divided into two groups, control air-exposed (Air, n = 18) and cigarette smoke exposed (CS, n = 18) groups. The mice in the CS group were exposed to cigarette smoke for 10 min inside an acrylic box twice a day, 4–5 times a week, for 4 months using 1 cigarette (Peace, Japan Tobacco Inc., Tokyo, Japan) for every exposure following a previous report with slight modifications^22^. The mice in the Air group were identically exposed to air without CS in a similar box. After the CS treatment, the animals were kept under normal warm and clean environmental conditions to allow for recovery and then housed as described above.

To evaluate the increase of inflammatory cells in airway and lungs, the lungs of mice were lavaged five times (1 ml × 5) through a tracheal cannula (n = 4 each). The bronchoalveolar lavage (BAL) was performed with 1 ml PBS. Cells were pelleted by centrifugation, and BAL cells were counted using a microscope. For differential cell counts, cytospin preparations were made and cells were fixed and stained with Giemsa Stain Solution (Nacalai Tesque).

To evaluate the progression of inflammation in the lungs, lung tissue homogenate of animals from the 2 groups (n = 4 each) was collected for western blot analysis. The excised lungs in Passive Lysis Buffer (Promega, WI, USA) were homogenized by homogenizer (T18 digital Ultra-Turrax, IKA Japan, Osaka, Japan) and centrifuged to remove debris, and subsequently diluted in dithiothreitol containing sample buffer (1.5 mg/ml). The samples (2 μl) were then loaded and western blotting was performed in accordance with the manufacturer’s protocol using anti-mouse MMP-9 (ab38898, Abcam, Cambridge, UK) and MMP-12 (ab66157, Abcam) primary antibodies and horseradish peroxidase-conjugated as a secondary antibody (HAF008, R&D Systems) to detect the MMPs. β-actin levels were used to control for protein loading in the samples, and were measured with anti-β-actin antibody (NB600-505SS, Novus Biologicals, CO, USA). Immunoreactive bands were visualized by Chemi-Lumi One L (Nacalai Tesque). A molecular weight marker (Broad Range, Nacalai Tesque) was used to estimate molecular weight, and the bands were observed using a ImageQuant LAS 4000 Mini (GE Healthcare Japan, Tokyo, Japan).

### ***In Vivo*** Biodistribution Study

Animals from each study group were randomly divided into 3 groups (n = 3 each) for each time point studied. Mice that were intravenously administered ^18^F-IPFP (185 kBq/100 μl saline containing 0.1% tween 80) were sacrificed by cervical dislocation at 20, 45, and 90 min, and 5, 45, and 90 min after administration for the Air and CS groups, respectively. Blood was immediately collected from the heart cavity. Tissues were excised, weighed, and counted for radioactivity with an NaI well-type scintillation counter (1470 Wizard; PerkinElmer, Kanagawa, Japan).

### PET Experiments

PET data were acquired in list mode using a microPET Focus120 apparatus (Siemens), with a spatial resolution of 1.2 mm at the center of the field of view^24^. Animals (n = 2 per group) were placed in a prone position under 1–2% isoflurane anesthesia on a heating pad to maintain body temperature at 36–37 °C during the PET experiments. A transmission CT scan using an external ^68^Ge source was acquired before the PET scan for attenuation correction. At the start of the PET scan, intravenous administration of ^18^F-IPFP (4.7–8.1 MBq/100 μl saline containing 0.1% tween 80) was initiated for 15 sec followed by a 50–100 μl saline flush for 15 sec. The scan duration was 60 min. Following the PET scan, mice were immediately euthanized with an anesthesia overdose, followed by extraction of the lungs and heart. Extracted organs were placed on the PET bed and scanned for another 10 min. Thereafter, the lungs were frozen for *ex vivo* autoradiography analysis. Emission images were reconstructed using Fourier rebinning^25^ and a maximum likelihood expectation maximization algorithm with a 128 × 128 × 95 matrix size, and a 0.43 × 0.43 × 0.80 mm^3^ voxel size. The uptake of the tracer in the regions of interest was converted to the standardized uptake values (SUVs).

### ***Ex Vivo*** Autoradiography and Immunohistochemistry

Following the PET experiments, the frozen lungs were cut into 20 μm thick sections with a cryomicrotome (Cryotome FSE; ThermoFisher Scientific, Tokyo, Japan). Lung sections at intervals of 100 μm (n = 2 each, 9 positions) were exposed to imaging plates (BAS-SR; GE Healthcare Japan, Tokyo, Japan) for 3 h. To quantify the signals, various concentrations of the administrated ^18^F-IPFP dilutions were also plotted and exposed to imaging plates. Autoradiograms were obtained with a Typhoon FLA 7000 scanner, (GE Healthcare Japan), and the resulting images were analyzed by ImageQuant TL (GE Healthcare Japan). Radioactivities per area were determined by image analysis of the resulting autoradiograms and expressed as percent injected dose (%ID) per section calculated using the quantitation curve obtained by the ^18^F-IPFP dilutions.

The adjacent sections were fixed by acetone (−20 °C) for 10 min followed by blocking using Blocking One Histo (Nacalai Tesque) for 10 min. Primary antibodies (ab38898 and ab66157) were diluted in PBS(−) with 1.5% BSA (10 μg/mL) and incubated overnight at 4 °C. After rinse, Alexa488 conjugated secondary antibody in PBS(−) (SAB4600044 2 μg/mL, Sigma, MO, USA) was incubated for 1 hr. Fluorescence images were acquired by fluorescence microscopy (BIOREVO BZ9000, Keyence Japan Co., Osaka, Japan) and fluorescence images were analyzed with BZ-II Analyzer 1.10 software (Keyence Japan Co.).

### Statistics

Data were expressed as means ± standard deviations. Statistical significance was assayed with the Mann-Whitney U test using GraphPad Prism 6. A *p* value < 0.05 was considered statistically significant unless otherwise noted.

### Data Availability

All data generated or analyzed during this study are included in this published article (and its Supplementary Information files).

## Electronic supplementary material

Supplementary information
