# Supplementary material for: Development of matrix metalloproteinase-targeted probes for lung inflammation detection with positron emission tomography
Source: Sci Rep. 2018 Jan 22;8:1347. doi: 10.1038/s41598-018-19890-1 (PMC5778071; doi:10.1038/s41598-018-19890-1)
Supplement: Supplementary file 1 — Supplementary information [file 41598_2018_19890_MOESM1_ESM.pdf]

Supplementary Information

Title:
Development of matrix metalloproteinase-targeted probes for lung inflammation detection with positron emission tomography

Authors:
Naoya Kondo, Takashi Temma*, Kazuki Aita, Saeka Shimochi, Kazuhiro Koshino, Michio Senda, Hidehiro Iida

Supplemental Scheme 1

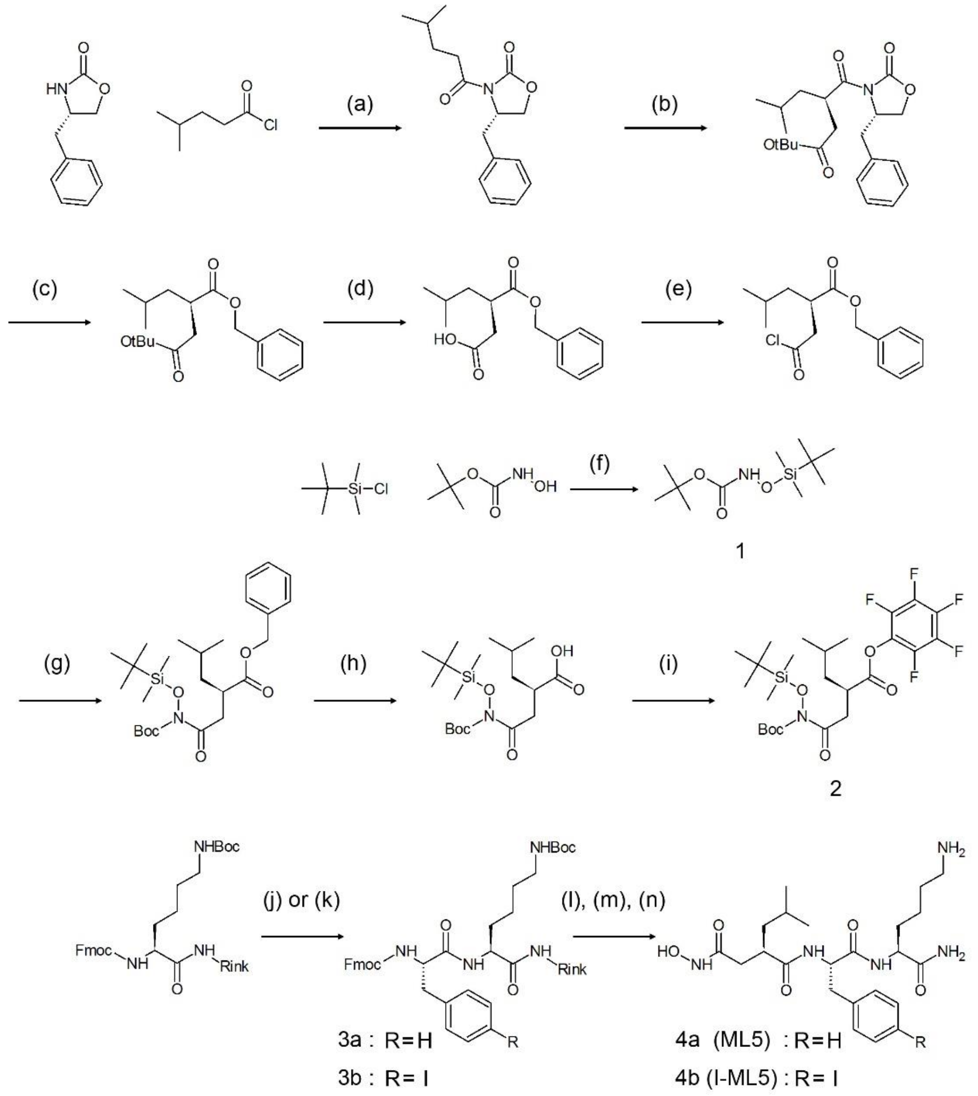

(a) 1.2 eq. *n*-BuLi, THF, -78°C; (b) 1.2 eq. LiHMDS, *t*-Butyl-Bromoacetate, THF, -78°C; (c) 2 eq. BnOH, 1.2 eq. *n*-BuLi, THF, 0°C; (d) TFA, DCM, RT; (e) 5eq. $(COCl)_2$, cat. DMF, DCM, 0°C; (f) Et_3N, DCM, 0°C; (g) 1.1 eq. **1**, 2 eq. DMAP, acetonitrile, 0°C; (h) H_2, Pd/C, MeOH; (i) 1 eq. PFP-OH, 1 eq. EDC, DCM, RT; (j) 5 eq. Fmoc-Phe-OH, HOBt, DIEA, DIPCI, RT; (k) 5 eq. Fmoc-Phe(4-I)-OH, HOBt, DIEA, DIPCI, RT; (l) 25% piperidine/DMF; (m) 3 eq. **2**, 2% DIEA/DMF; (n) 95% TFA/H_2O.

2 Pentafluorophenyl (2-(R)-isobutyl-3-(*N*-tert-butoxycarbonyl-*N*-(tert-butyl-dimethylsilyloxy)-carbamoyl) propionate); MS (ESI) *m/z* 570.3 $[M+H]^+$ (Chemical formula: $C_{25}H_{36}F_5NO_6Si$, calculated *m/z* value: 569.2). ^1H NMR (400 MHz, $CDCl_3$): δ 3.37-3.28 (m, 2H), 3.11-3.03 (m, 1H), 1.82-1.70 (m, 2H), 1.56 (s, 9H), 1.53-1.46 (m, 1H), 1.00 (d, *J* = 6.4 Hz, 3H), 0.99 (s, 9H), 0.95 (d, *J* = 6.0 Hz, 3H), 0.13, (s,

3H), 0.12 (s, 3H).

Supplemental Scheme 2

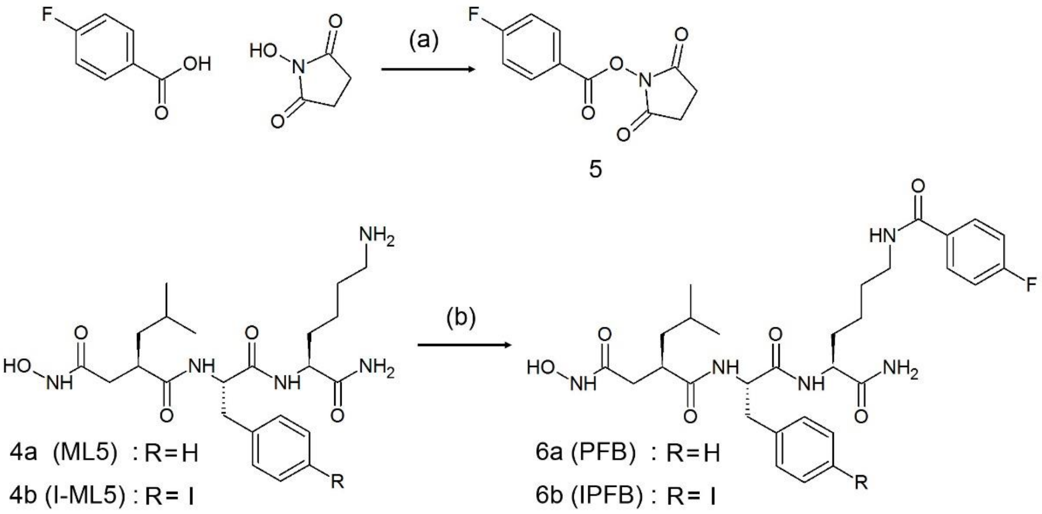

(a) 1.1 eq. *N*-hydroxy succinimide, DCM, DMF, 0°C; (b) 1 eq. **5**, phosphate buffer (pH 8.5)/acetonitrile = 1:1, 50°C, 30 min.

5 SFB; MS (ESI) *m/z* 238.2 $[M+H]^+$ (Chemical formula: $C_{11}H_8FNO_4$, calculated *m/z* value: 237.0). ^{1}H NMR (400 MHz, $CDCl_3$): δ 8.20-8.15 (m, 2H), 7.23-7.17 (m, 2H), 2.92 (s, 4H).

Supplemental Scheme 3

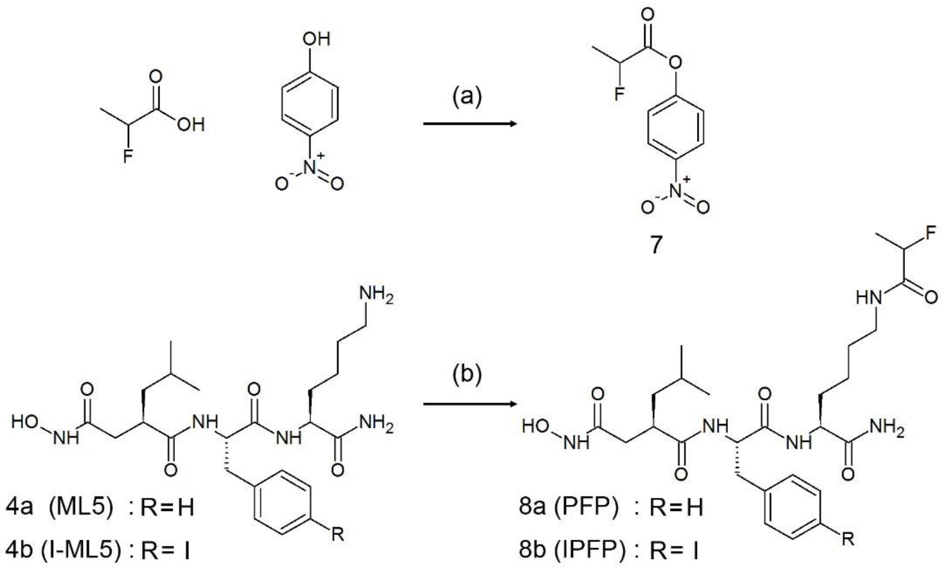

(a) 5 eq. $(COCl)_2$, cat. DMF, DCM, 0°C then, 2 eq. DMAP, acetonitrile, 0°C; (b) 1 eq. **7**, phosphate buffer (pH 8.5)/acetonitrile = 1:1, 50°C, 30 min.

7 NFP; MS (ESI) *m/z* 214.2 $[M+H]^+$ (Chemical formula: $C_9H_8FNO_4$, calculated *m/z* value: 213.0). ^1H NMR (400 MHz, $CDCl_3$): δ 8.32-8.29 (m, 2H), 7.37-7.33 (m, 2H), 5.37-5.20 (dq, *J*= 48.0, 6.8 Hz, 1H), 1.77 (dd, *J*= 23.6, 7.2 Hz, 3H).

8b IPFP; MS (ESI) *m/z* 664.3 $[M+H]^+$ (Chemical formula: $C_{26}H_{39}FIN_5O_6$, calculated *m/z* value:663.2). ^1H NMR (600 MHz, CD_3OD): 7.61 (d, *J*= 8.1 Hz, 2H), 7.05 (d, *J*= 8.1 Hz, 2H), 4.96 (dq, *J*= 48.9, 6.8 Hz, 1H), 4.57 (dd, *J*= 9.6, 5.7 Hz, 1H), 4.28 (m, 1H), 3.22 (m, 2H), 3.19 (m, 1H), 2.94 (dd, *J*= 14.0, 9.6 Hz, 1H), 2.69 (m, 1H), 2.20 (dd, *J*= 14.7, 8.3 Hz, 1H), 2.09 (dd, *J*= 14.7, 6.3 Hz, 1H), 1.81 (m, 1H), 1.71 (m, 1H), 1.54 (m, 2H), 1.50 (dd, *J*= 23.1, 6.8 Hz, 3H), 1.41 (m, 1H), 1.40 (m, 2H), 1.26 (m, 1H), 1.08 (m, 1H), 0.83 (d, *J*= 6.6 Hz, 3H), δ0.79 (d, *J*= 6.5 Hz, 3H).

Supplemental Scheme 4

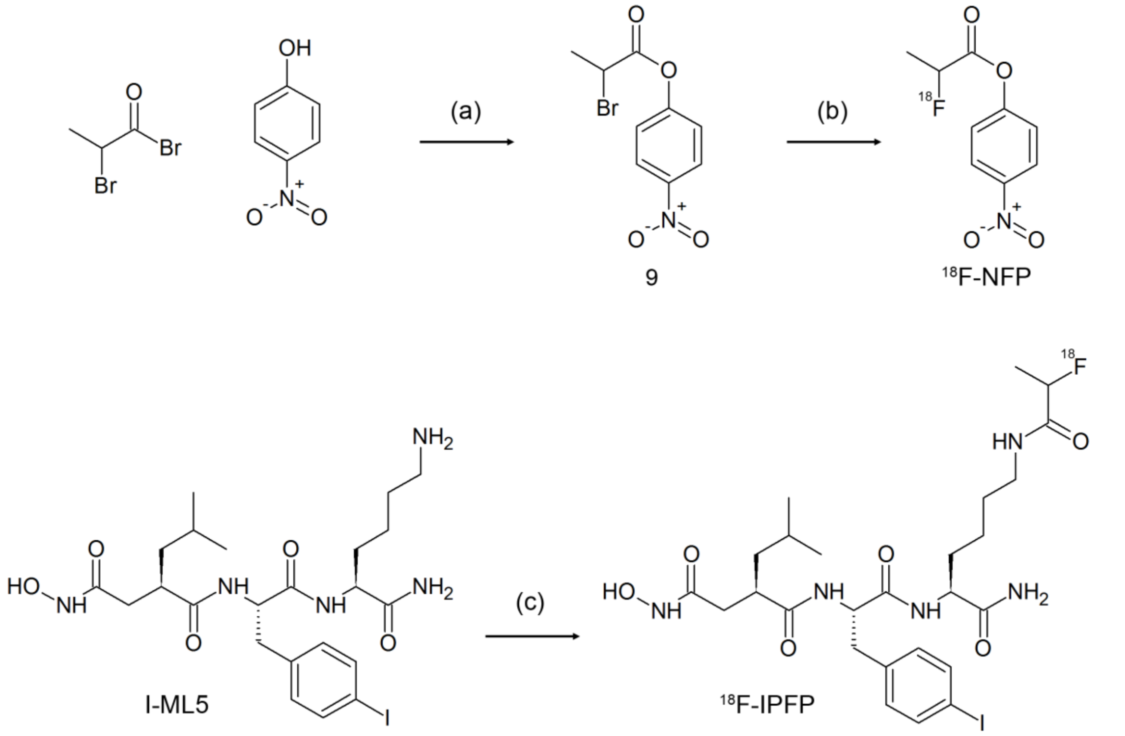

(a) Et_3N, DCM, 0°C, 1 hr; (b) Fluorine-18 was produced as an ^{18}F-fluoride ion via the ^{18}O(p,n)^{18}F reaction in ^{18}O-water using an in-house CYPRIS-HM18 cyclotron (Sumitomo Heavy Industries, Ltd., Tokyo, Japan). ^{18}F-Fluoride was trapped by a QMA cartridge (Waters, Tokyo, Japan) and eluted to the reactor vial with 1 ml of acetonitrile/water (85/15) solution containing potassium bicarbonate (5 mg) and Kryptofix 222 (13 mg). The solution was azeotropically dried with anhydrous acetonitrile at 140˚C. To the anhydrous residue, 4-nitrophenyl 2-bromopropionate 9 (20 mg, 0.05 mmol) in tBuOH- acetonitrile (4:1, 2 mL) was added. After 5 min at 100˚C, ^{18}F-NFP was purified by preparative HPLC. ^{18}F-NFP was synthesized using an automated JFE multi-purpose synthesizer; (c) ^{18}F-NFP was concentrated (100–300 MBq/200 μL acetonitrile) and transferred to a solution of I-ML5 (500 μg) in 200 μL phosphate buffer (pH 8.5) and reacted at 50˚C for 30 min.

Supplemental Table 1: Characteristics of the synthesized compounds

Compound	[a]ESI-MS (*m/z*)		[b]Retention time	Yield
	Calculated	*Found* $[M+H]^+$	(min)	(%)
ML5	463.3	464.4	7.4	[c]20
I-ML5	589.2	590.3	9.4	[d]25
PFB	585.3	586.4	9.9	[e]15
IPFB	711.2	712.3	12.0	[f]35
PFP	537.3	538.4	9.9	[e]12
IPFP	663.2	664.3	12.0	[f]30

(a) Mass spectra were obtained as *m/z* with an LCMS-2020 (Shimadzu, Kyoto, Japan).

(b) RP-HPLC was performed with a Shimadzu system comprised of an LC-20AT pump with an SPD-20A UV detector (λ = 254 nm), using a Cosmosil C18 column (Nacalai Tesque, 5C18-AR-II, 10 × 250 mm). The mobile phase [0.1% trifluoroacetic acid (TFA) in water/0.1% TFA in acetonitrile = 90/10 (0 min) to 10/90 (20 min)] was delivered at a flow rate of 5.0 mL/min.

(c) Calculated from dipeptide “3a”.

(d) Calculated from dipeptide “3b”.

(e) Calculated from ML5.

(f) Calculated from I-ML5.

Supplemental Figure 1: Co-elution of ^{18}F-IPFP and reference standard.

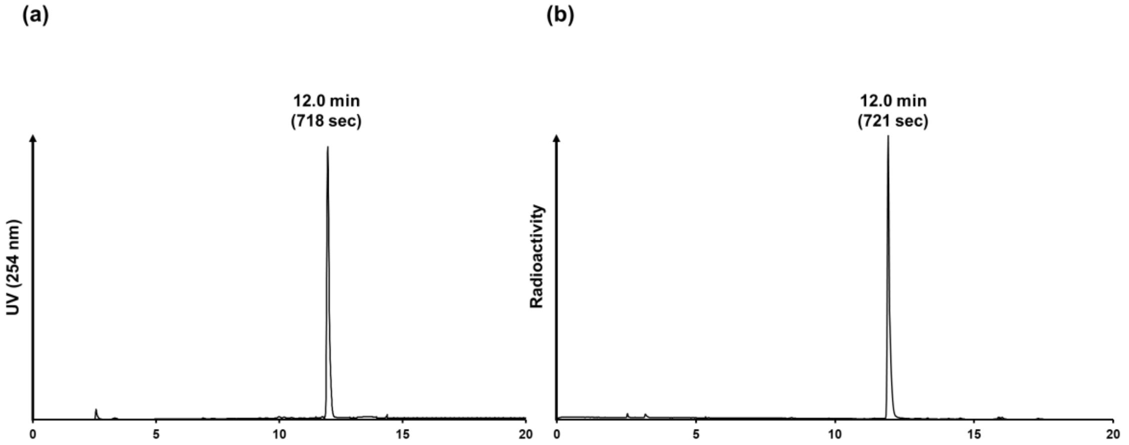

Co-elution of ^{18}F-IPFP and reference standard detected with (a) UV (λ = 254 nm) and (b) radioactivity using a Cosmosil C18 column (Nacalai Tesque, 5C18-AR-II, 10 × 250 mm). The mobile phase [0.1% trifluoroacetic acid (TFA) in water/0.1% TFA in acetonitrile = 90/10 (0 min) to 10/90 (20 min)] was delivered at a flow rate of 5.0 mL/min.

Supplemental Figure 2.

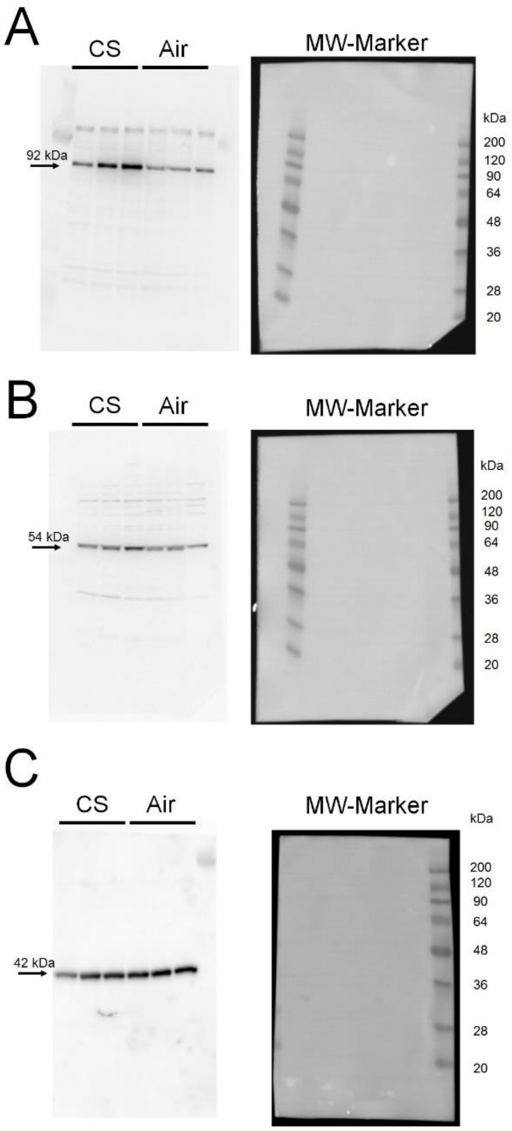

Representative western blot bands: (A) MMP-9, (B) MMP-12 and (C) β-actin in lung homogenates of the CS and Air groups with MW-marker in full-length gels.
